# Combining Geostatistics and Remote Sensing Data to Improve Spatiotemporal Analysis of Precipitation

**DOI:** 10.3390/s21093132

**Published:** 2021-04-30

**Authors:** Emmanouil A. Varouchakis, Anna Kamińska-Chuchmała, Grzegorz Kowalik, Katerina Spanoudaki, Manuel Graña

**Affiliations:** 1 School of Environmental Engineering, Technical University of Crete, 73100 Chania, Greece; 2 Department of Computer Science and Systems Engineering, Wroclaw University of Science and Technology, 50-370 Wroclaw, Poland; grzegorzkow95@gmail.com; 3 Institute of Applied and Computational Mathematics, Foundation for Research and Technology, GR-700 13 Heraklion, Greece; kspanoudaki@gmail.com; 4 Computational Intelligence Group, Computer Science Faculty, University of the Basque Country, UPV/EHU, 00685 San Sebastián, Spain; manuel.grana@ehu.es

**Keywords:** satellite data, geostatistics, space–time residual kriging, machine learning, sum-metric

## Abstract

The wide availability of satellite data from many distributors in different domains of science has provided the opportunity for the development of new and improved methodologies to aid the analysis of environmental problems and to support more reliable estimations and forecasts. Moreover, the rapid development of specialized technologies in satellite instruments provides the opportunity to obtain a wide spectrum of various measurements. The purpose of this research is to use publicly available remote sensing product data computed from geostationary, polar and near-polar satellites and radar to improve space–time modeling and prediction of precipitation on Crete island in Greece. The proposed space–time kriging method carries out the fusion of remote sensing data with data from ground stations that monitor precipitation during the hydrological period 2009/10–2017/18. Precipitation observations are useful for water resources, flood and drought management studies. However, monitoring stations are usually sparse in regions with complex terrain, are clustered in valleys, and often have missing data. Satellite precipitation data are an attractive alternative to observations. The fusion of the datasets in terms of the space–time residual kriging method exploits the auxiliary satellite information and aids in the accurate and reliable estimation of precipitation rates at ungauged locations. In addition, it represents an alternative option for the improved modeling of precipitation variations in space and time. The obtained results were compared with the outcomes of similar works in the study area.

## 1. Introduction

Nowadays, remote sensors such as satellites located in a geostationary, medium or low Earth orbit have multiple applications in innovative research and monitoring Earth processes. Additionally, Light Detection and Ranging (LiDAR) sensors or other radars also have been applied in a wide spectrum of disciplines, e.g., in environmental studies to support leaf area index (LAI) estimation by using LiDAR height and intensity data [1], in traffic emission, and to estimate vehicle speeds [2] or to generate more detailed 3D building models from LiDAR scanning [3]. The accuracy of the measurements gives us the opportunity to achieve a global coverage with a resolution of up to several dozen centimeters. This is possible thanks to increasingly effective instruments mounted in the next series of satellites, caused by the dynamic development of technology and permanent investments in the space industry. Satellite data have a wide range of applications in domains such as forestry, agriculture, land management, object detection, military, environment, geology, etc. One of the most important areas of application is hydrology. Based on information from satellite instruments and enhanced by artificial intelligence (AI) algorithms, it is possible to extract information and to calculate precipitation rates in areas of interest [4,5,6]. AI and machine learning development have improved the processing of large datasets of different input variables from remote sensors, very quickly and efficiently, in order to provide output products in areas such as precipitation and evapotranspiration, in spatial and temporal resolution suitable for applications in hydrology and geosciences [5,7].

The Mediterranean region is the region over the Mediterranean Sea and extends over three continents, Europe, Asia and Africa. It can be viewed as a transitional zone located between the moderate climate of European mid latitudes and the semi-arid and arid climate of northern Africa and the Middle East [8,9]. Many studies have been published about precipitation in the Mediterranean region, which has been affected by climatic variability. During the past decades, precipitation spatiotemporal variability has been altered in the Mediterranean region and has a decreasing tendency. Particularly in the south-eastern Mediterranean region, where Crete is located, precipitation is an important parameter for the sustainability of water resources, which has been affected by the variations in precipitation caused by climatic variability. The precipitation duration has become shorter and the intensity has increased, causing more frequent flooding events [9,10,11,12,13,14,15,16].

The Mediterranean climate is overall defined as a mid-latitude temperate climate and is characterized by rainy winters and hot, dry summers. In different regions of the Mediterranean, the winter and summer temperatures can vary greatly. High rates of evapotranspiration are observed while the climate varies greatly at multiple timescales, and significant heterogeneity of strong seasonal variability in precipitation regimes occurs in many areas [8]. In the eastern Mediterranean zone in particular, rainfall variability is caused by anomalies associated with North Atlantic climate variability [17].

In Greece, there is also a strong difference between the rainier western Greek region and the drier eastern part, while the islands, particularly in the southern parts, experience low precipitation rates. In southern Greece, the contribution of winter precipitation to the annual totals exceeds 50%, and summer is the dry season [18].

In contrast to a known decreasing trend in the southern parts of Greece, an increasing trend in precipitation has been observed in the central and northern parts of the country. [19]. On the other hand, a decreasing tendency in winter rainfall in Greece has been observed, while the decreasing trend during autumn and spring is considered less significant [20]. In addition, in Greece, a significant negative correlation has been found between the North Atlantic Oscillation Index (NAOI) and precipitation variability [19].

This research work focuses on the Greek island of Crete, which is located in the southeastern part of the Mediterranean Sea. Crete was selected as a study area due to the availability of precipitation data and because, according to recent studies, it is a hot spot for the effects of climate change [21,22]. Rainfall variability in Crete was also found to be linked to NAOI variability. For the period from 1981 to 2015, there was a statistically significant negative association between the island’s rainfall variability and the North Atlantic Oscillation Index [23].

The present work introduces a geostatistical methodology based on the space–time kriging method that combines satellite and ground precipitation observations exploiting the spatiotemporal interdependence of space–time data for the enhancement of spatiotemporal mapping and analysis, both in terms of spatial resolution and accuracy. The extension of the methodology that incorporates auxiliary information is known as space–time regression or residual kriging (STRK). In this study, the covariates were the post-processed PERSIANN-CCS satellite precipitation data and geographical features. Space–time kriging and STRK have been successfully applied to different datasets and rainfall networks to analyze precipitation variations in space and time [24,25,26,27,28,29,30,31,32,33,34]. These works endorse the applicability of the methodology on precipitation data. Space–time geostatistics can be useful in exploiting the interdependence of the data and in incorporating auxiliary variables in order to provide reliable estimations in ungauged locations. In addition, it is a useful tool to estimate precipitation in higher spatial resolution compared to the available satellite products, which exploit denser geographical features as auxiliary information.

The fusion of satellite and ground precipitation information has been applied mainly in spatial or temporal only geostatistical analysis [35,36], and very rarely in spatiotemporal geostatistical analysis [13,37,38]. However, in this work, a new extension of the methodology is applied, which incorporates auxiliary information of satellite precipitation observations and geographical covariates to improve precipitation mapping. In addition, another novelty is the successful assessment of the sum-metric space–time variogram model in such an approach. STRK has been applied previously in Crete [13], but this work applies a trend model that considers different input information.

This study considers annual precipitation data for the hydrological period from 2009/10 to 2017/18. The hydrological year in the Mediterranean region starts in October and ends in September of the following year. The prediction performance of the proposed method is tested by comparing predictions with the available data for the hydrological year 2016/17, which recorded an average annual precipitation rate close to the historical average. A recent year was selected because it follows two hydrological years of extreme high and low precipitation rates, which allows the geostatistical model to be trained with the extreme behavior of the study variable in the region.

## 2. Methodology

### 2.1. Study Area and Data

**Figure 1:**
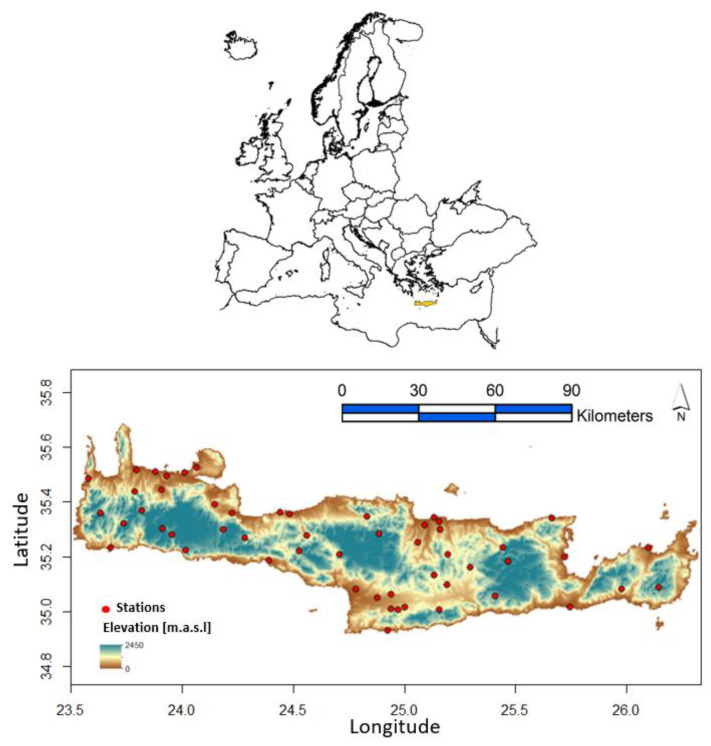
Digital elevation map of Crete providing the locations of the 53 meteorological stations.

The island of Crete has a dry sub-humid Mediterranean climate. Precipitation rates over the island vary according to the distance from the sea and the altitude difference, while a precipitation gradient exists from the east to the west of the island. The average annual precipitation in Crete has been reported historically to be around 927 mm [39,40]. However, according to a new report covering the years from 1981 to 2014, the total annual rainfall on the island was 798.3 mm [23]. In the island of Crete, a monitoring network of 53 randomly distributed meteorological stations is operated from the National Observatory of Greece (Figure 1) to cover the entire island area and its variable topography. For this work, precipitation measurements were received and processed from these stations. The data consist of annual precipitation rates during the 2009/10–2017/18 period [41]. Auxiliary correlated information in terms of geographical covariates is also used to support the space–time geostatistical analysis of precipitation in the island of Crete. The terrain variability affects the precipitation patterns [42,43,44]. However, in this work, any terrain effect is embedded in the spatiotemporal model through the ground precipitation observations that are affected from elevation variability, meaning that it is summarized in the spatiotemporal variogram. Consideration of the complexity of the terrain should be dealt with by non-stationary methods, which model the local spatial complexity, while our method assumes stationarity, that is, homogeneous terrain complexity [33,44].

### 2.2. Satellite Sensors Data

**Figure 2:**
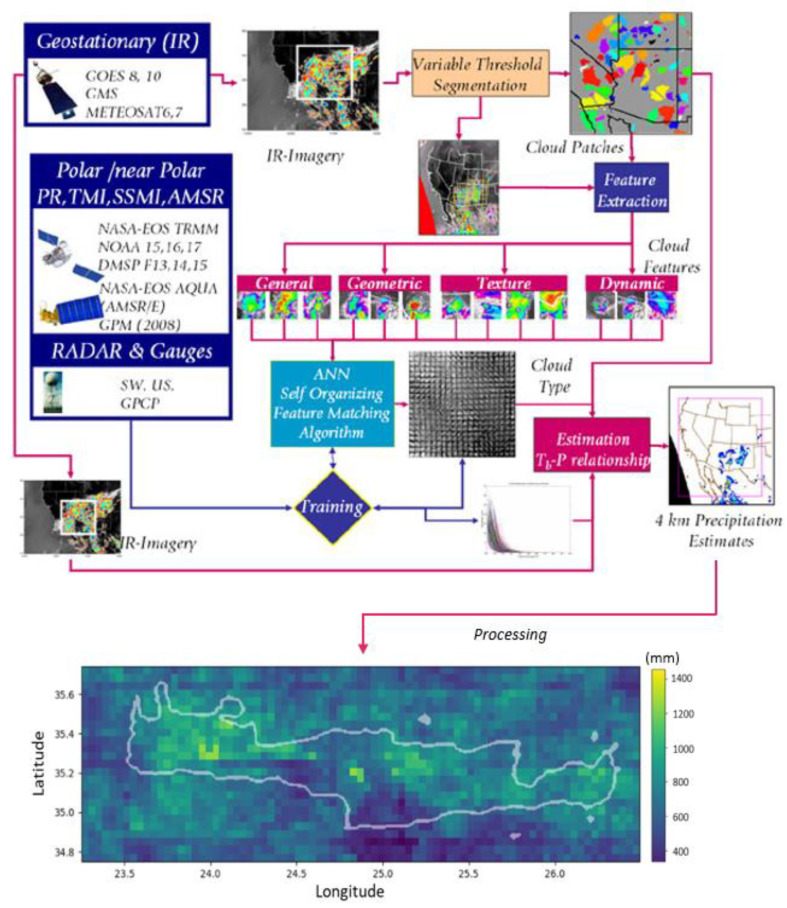
Data-processing pipeline (adapted from: http://chrs.web.uci.edu/SP_activities01.php, accessed on 1 April 2021). Average annual precipitation (mm) over the island of Crete for the study period 2009/10–2017/18 obtained from the PERSIANN-CCS satellite database.

The satellite data covering the period between the hydrological years 2009/10 and 2017/18 are obtained from the PERSIANN Cloud Classification System (PERSIANN-CCS) developed by the Center for Hydrometeorology and Remote Sensing (CHRS) at the University of California, Irvine (UCI). The PERSIANN-CCS precipitation estimates are computed based on the categorization of cloud features, extracted from satellite images. It uses a variable threshold cloud segmentation algorithm, which provides better cloud segmentation accuracy in comparison to traditional approaches, by means of constant threshold segmentation. The separated cloud patches are then classified based on their features, which helps in assigning a rainfall value to each pixel in each cloud. For cloud segmentation and classification, long-wave infrared (10.2–11.2 um) measurements from geostationary satellites are processed. To achieve worldwide coverage, images from GOES, GMS and METEOSAT satellites are used. The data-processing pipeline is shown in Figure 2. For assigning rainfall estimation values, the system uses a neural network trained on a vast variety of data sources, such as NASA-EOS TRMM and AQUA, NOAA, DSMP and GPM satellites, but also radar cloud reflectivity and rain gauge measurements. The detailed description of how clouds are classified and how rainfall values are estimated is presented in [6]. Precipitation estimates calculated using the PERSIANN-CCS are freely available on the CHRS Data Portal [5]. The dataset provides global coverage, with some exceptions, with spatial resolution of 0.04 degrees and time resolution of an hour. The data available on the CHRS portal spans from January 2003 to the present day. Data from the Data Portal was processed, converted to pixels and aggregated using Python scripts implemented by authors to create an entry dataset for further processing (Figure 2). Figure 2 presents the output average satellite estimates in the area of Crete for the study period, created after an aggregation of the annual values. Spatial and temporal resolutions of the final dataset are the same as CHRS data. The assumption that the exported precipitation is evenly distributed in each grid cell applies.

### 2.3. Space–Time Kriging Method

A spatiotemporal geostatistical analysis of precipitation measurements was performed using space–time regression or residual kriging (STRK). This methodology has been successfully applied in similar applications using annual precipitation data [28,31,33]. The geostatistical analysis using the proposed methods and tools was performed using the “gstat” package of R software [45]. Space–time observations in *N* sample points are defined as Z(s,t), where *Z* is the observation, **s** the spatial coordinates and *t* the time step, whereas the estimates in space–time depend on $Z(s_{i},t_{i})$, …, $Z(s_{N},t_{N})$. The observation values can be analyzed into two terms following Equation (1):

(1) $$Z(s,t)=\;\;m_{Z}(s,t)+Z^{\prime }(s,t)$$

where, $m_{Z}(s,t)$ is the trend term and $Z^{\prime }(s,t)$ the residual term modeling the space–time fluctuations. The trend term is usually calculated using correlated auxiliary variables. The space–time residual model involves: $m_{Z}(s,t)=\beta_{1}{Χ}_{1}\left(s\right)+\beta_{2}{Χ}_{2}\left(s\right)+\beta_{0}$, where β are the regression coefficients, and $\;{Χ}_{1}\left(s\right)\;$and ${Χ}_{2}\left(s\right)$ are covariates related to observed precipitation, where the former denotes the average satellite estimates for the study period and the latter the longitude direction of observation locations *s.* The latter is applied because from the initial analysis of data correlation between ground observations and satellite data, it was found that the correlation varies more in the longitude direction (62–81%) than in the latitude direction (69–76%). The overall correlation was 71%. Thus, the spatiotemporal random field of annual precipitation fluctuates along a spatiotemporal trend, which was removed using satellite precipitation data and longitude coordinate direction.

The experimental spatiotemporal variogram model of the residuals is given by:

(2) $${\hat{\gamma }}_{Z}(r_{s},r_{t})=\frac{1}{2N\left(r_{s},r_{t}\right)}\sum_{N\left(r_{s},r_{t}\right)}{\left[Z^{’}(s_{i},t_{i})-Z^{’}(s_{j}+r_{s},t_{j}+r_{t})\right]}^{2}$$

where $r_{s}$ = ||***s**_i_*− ***s***_*j*_||, $r_{t}$ = |*t_i_* − t_*j*_|, and *N*($r_{s},r_{t}$) is the number of space–time pairs in the corresponding lags.

The space–time kriging estimator using residual data notation is given below:

(3) $${\hat{Z}}^{\prime }\left(s{\;}_{0},t{\;}_{0}\right)=\sum_{\left\{i:\;s_{i},t_{i}\in S_{0}\right\}}\lambda_{\;i}\;Z^{\prime }\left(s{\;}_{i},t{\;}_{i}\right),$$

where $S_{0}$ denotes the observation points that belong in the estimation neighborhood of $\left(s{\;}_{0},t{\;}_{0}\right)$. The term ${\hat{Z}}^{\prime }\left(s{\;}_{0},t{\;}_{0}\right)$ denotes the estimation point in terms of location and time, $Z^{\prime }\left(s_{i},t{\;}_{i}\right)$ are the observed values/residuals of the neighborhood at specific location and time and $\lambda_{\;i}$ express the weights of the space–time kriging process determined as follows:

(4) $$\sum_{\left\{i:\;s_{i},t_{i}\in S_{0}\right\}}\lambda_{\;i}\;\gamma_{Z^{\prime }}\;(s_{i},s_{j};t_{i},t_{j})+\mu =\;\gamma_{Z^{\prime }}\;(s_{j},s_{0};t_{j},t_{0}),\;j=1,\ldots ,N_{0}$$

(5) $$\sum_{\left\{i:\;s_{i},t_{i}\in S_{0}\right\}}\lambda_{\;i}\;=1$$

$N_{0}$ is the number of points within the search neighborhood of $\left(s{\;}_{0},t{\;}_{0}\right)$, $\gamma_{Z^{\prime }}\;(s_{i},s_{j};t_{i},t_{j})$ is the variogram between two sampled points $s_{i}$ and $s_{j}$ at times $t_{i}$ and $t_{j}$, $\;\gamma_{Z^{\prime }}\;(s_{j},s_{0};t_{j},t_{0})$ the variogram between $s_{j}$, $t_{j}$ and the estimation point $s_{0}$, $t_{0}$ and μ is the Lagrange multiplier enforcing the zero bias constraint.

The STRK estimate of the precipitation rate is expressed as:

(6) $$\hat{Z}(s_{0},t_{0})=\;\;m_{Z}(s_{0},t_{0})+{\hat{Z}}^{\prime }(s_{0},t_{0})$$

where $\;m_{Z}(s_{0},t_{0})$ is the estimated trend function, and ${\hat{Z}}^{\prime }(s_{0},t_{0})$ is the interpolated residual computed by means of space–time ordinary kriging.

The variance of the STRK estimator is given by:

(7) $$Var\;\left(\hat{Z}\left(s_{0},t_{0}\right)-Z\left(s_{0},t_{0}\right)\right)\;\;\;\;\;\;\;\;\;\;\;\;\;\;\;\;\;\;\;\;\;\;\;\;\;\;\;\;\;\;\;\;\;\;\;\;\;\;\;\;\;\;\;\;\;\;\;\;\;\;\;\;\;\;\;\;\;\;\;\;\;\;\;\;\;\;\;\;\;\;\;\;\;\;\;\;\;\;\;\;\;=\sigma^{2}\left\{m_{Z}(s_{0},t_{0})\right\}+\left(\sum_{i=1}^{n}\lambda_{i}\cdot \gamma_{ST}\left(s_{i}-s_{0},t_{i}-t_{0}\right)+\mu \right)$$

where $\gamma_{ST}$ is the space–time (ST) variogram function in terms of the estimation point, while the variance of the estimated trend is given by:

(8) $$\sigma^{2}\left\{m_{Z}(s_{0},t_{0})\right\}={\left(x_{0}-X^{T}C^{-1}c_{0}\right)}^{T}{\left(X^{T}C^{-1}X\right)}^{-1}\left(x_{0}-X^{T}C^{-1}c_{0}\right)$$

**C** is the matrix of covariances at the observation coordinates, **X** is the measured covariates matrix at the observation coordinates, ***x***_o_ is the covariates vector at the estimation coordinates, and ***c***_o_ is the covariances vector between the observation and estimation points. The calculated kriging-variance corresponds to the associated uncertainty of estimations.

The space–time experimental variogram (2) is modelled using the sum-metric space–time variogram as well as the product-sum space–time variogram functions. The sum-metric model is specified by the following function [46,47]:

(9) $$\gamma_{ST}\left(r_{s},r_{t}\right)=\gamma_{ST}\left(r_{s},0\right)+\gamma_{ST}\left(0,r_{t}\right)+\gamma_{ST}\left(h\right),\;h=\sqrt{{r_{s}}^{2}+\alpha \;{r_{t}}^{2}}$$

where $\gamma_{ST}\left(r_{s},0\right)$, $\gamma_{ST}\left(0,r_{t}\right)$ and $\gamma_{ST}\left(h\right)$ are spatial, temporal and joint spatiotemporal variograms, while α is an anisotropic ratio parameter that approximates the space–time scale variance.

The product-sum model is formed by employing a fusion of a separate product and sum models [48]. Its covariance form is expressed as:

(10) $$C_{ST}\left(r_{s},r_{t}\right)=k_{1}C_{S}\left(r_{s}\right)C_{T}\left(r_{t}\right)+k_{2}C_{S}\left(r_{s}\right)+k_{3}C_{T}\left(r_{t}\right)$$

where $C_{S},C_{T}$ are space and time-oriented covariance models with $k_{1}>0$, $k_{2}\geq 0$, $k_{3}\geq 0$. In terms of the variogram, a non-separable function is formed [49] according to the following equation:

(11) $$\gamma_{ST}\left(r_{s},r_{t}\right)=\left(k_{1}C_{S}\left(0\right)+k_{3}\right)\gamma_{T}\left(r_{t}\right)+\left(k_{1}C_{T}\left(0\right)+k_{2}\right)\gamma_{s}\left(r_{s}\right)-k_{1}\gamma_{S}\left(r_{s}\right)\gamma_{T}\left(r_{t}\right)$$

where $\gamma_{S},\gamma_{T}$ are purely spatial and temporal variogram models.

In the present work, different variogram model types were assessed for the different terms of the sum-metric and product-sum space–time variograms through cross validation to determine the best fitted space–time model. The sum-metric was applied by means of a spatial Gaussian, a temporal exponential and a joint exponential model and the product sum by a spatial Gaussian and a temporal exponential model:

(12) $$\gamma_{z_{s}}(r)=\sigma {\;}_{z_{s}}^{2}\;\left[1-\exp{\left(-\frac{\left|r_{s}\right|}{\xi_{s}}\right)}^{2}\right]+c\;$$

(13) $$\gamma_{z_{t}}(r_{t})=\sigma {\;}_{z_{t}}^{2}\;\left[1-\exp\left(-\frac{\left|r_{t}\right|}{\xi_{t}}\right)\right]\;$$

(14) $$\gamma_{Z}(\sqrt{{r_{s}}^{2}+\alpha \;{r_{t}}^{2}})=\sigma {\;}_{Z}^{2}\;\left[1-\exp\left(-\frac{\sqrt{{r_{s}}^{2}+\alpha \;{r_{t}}^{2}}}{\xi_{J}}\right)\right]$$

where *σ* is the variance, *c* is the nugget variance term, ξ is the correlation length in space (*s*) and time (*t*), $\xi_{J}$ the joint scale (or range) parameter, and r is the lag vector in space and time.

## 3. Results and Discussion

**Figure 3:**
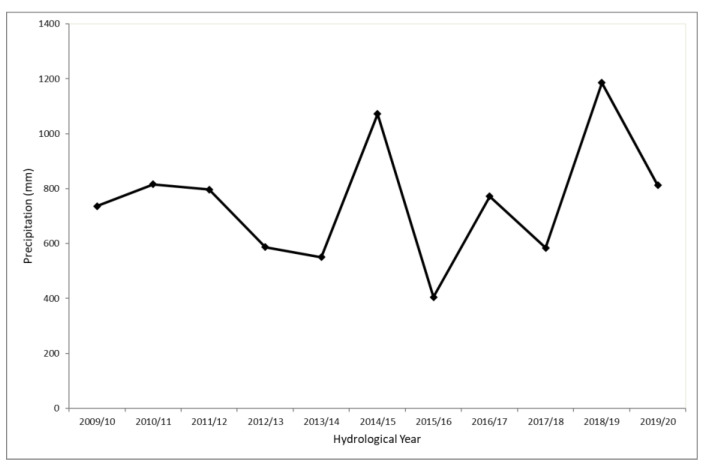
Annual average precipitation variation during the period 2009/10–2019/20.

The cumulative annual spatial average variation of precipitation in the island of Crete based on the ground observations is presented in Figure 3. The spatiotemporal trend of the ground precipitation observations was removed using satellite precipitation data and the location (easting direction) of the ground measurements. Next, the geostatistical analysis was carried out, which involved calculating the space–time experimental variogram of residuals, performing theoretical space–time variogram modelling and leave-one-out cross-validation, and finally applying the space–time kriging estimation of the residuals, incorporating the estimated trend surface at the estimation locations. The STRK residuals form a weakly stationary random field [50], and such an approach is usually applied to approximate non-stationarity issues that arise in precipitation data [51,52].

**Figure 4:**
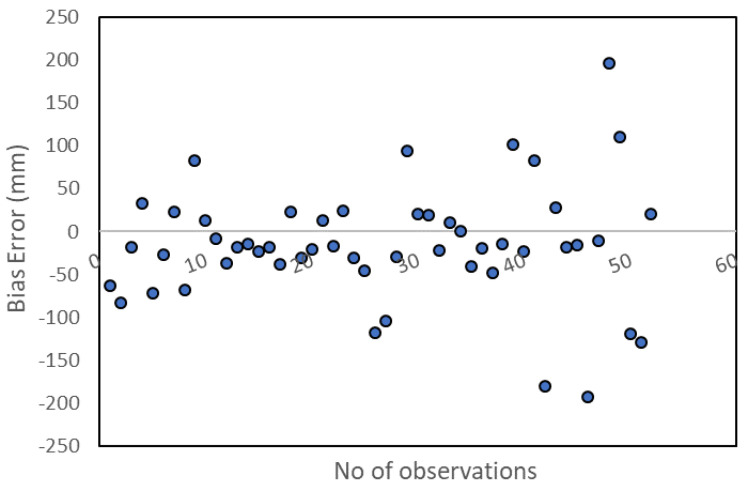
Bias performance of STRK method for the hydrological year 2016/17.

A full error analysis of the method’s capacity to estimate the annual precipitation values of the hydrological year 2016/17 at the 53 observation locations is presented in Table 1. As it can be observed by the estimation of metrics, the spatiotemporal residual kriging model that involves the sum-metric function delivers more accurate results compared to that which involves the product-sum. The difference might not be significant between the two variogram functions, but any enhancement is important for the improvement of the spatiotemporal variability estimation of physical variables. The bias scatterplot (Figure 4) provides the error distribution among the estimated values using STRK and the sum-metric function.

The sum-metric variogram parameters are presented in Table 2, while the product-sum variogram model parameters are as follows: $\sigma_{z_{s}}^{2}=42\;\;{\mathrm{mm}}^{2}$, $\xi_{\tau }=3.5\;\mathrm{yr}$, $\xi_{r}=0.31\;\mathrm{or}\;81\;\mathrm{km}$, the nugget variance $c=8.01\;{\mathrm{mm}}^{2}$ $\sigma_{z_{t}}^{2}=43.95\;\;{\mathrm{mm}}^{2}$, $k_{1}>0.95$, $k_{2}\geq 0.64$, $k_{3}\geq 1.12$.

**Figure 5:**
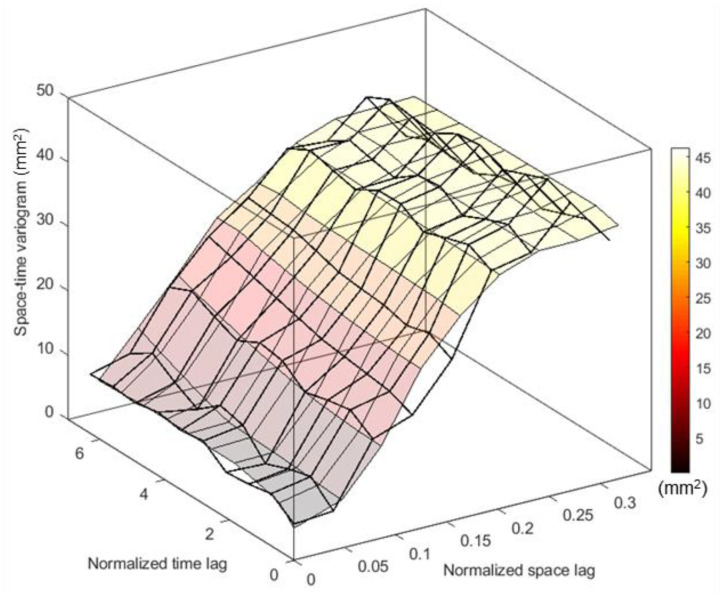
Spatiotemporal variogram fit of the theoretical sum-metric model (colored surface) to the experimental (black line grid).

The best performed sum-metric model fit is presented in Figure 5. A small decrease in the experimental space–time variogram is observed at separation distances around 0.32 units of normalized distance (approximately 76 km). This indicates that locations separated by 0.32 units are more similar than locations separated by shorter distances. This is explained by the location of the monitoring gauges in lowland areas of the island. Although, in the island, an east–west precipitation gradient exists, the lowland areas around the island receive similar rainfall values. These gauges are almost the half of the ensemble, and they are located around the entire island extend. In shorter distances, the geomorphology of the island affects the rainfall values extent and distribution. At short distances, there is similarity, which is normal variogram behavior, while at medium to longer distances the dissimilarity exists because of the variable geomorphology and the sparse dataset. More observations in space and time would provide different semivariance distribution of these lags.

**Figure 6:**
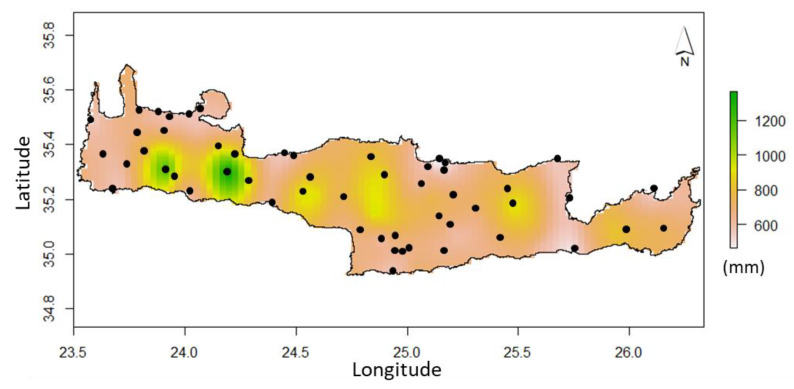
Spatial distribution of the estimated precipitation using STRK and the sum-metric space–time variogram model for the year 2016/17.

**Figure 7:**
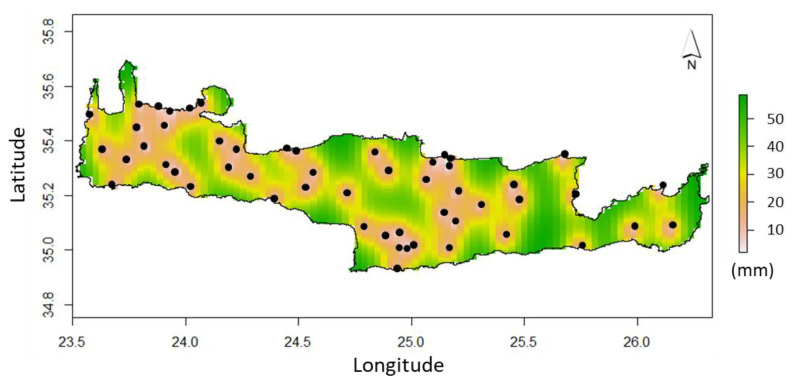
Spatial distribution of the estimated uncertainty using STRK and the sum-metric space–time variogram model for the year 2016/17.

The spatial distribution of the estimated precipitation in the entire island of Crete, associated with the uncertainty of estimations for the hydrological year 2016/17 based on the spatiotemporal dynamic behavior of the precipitation fluctuations in terms of their spatiotemporal interdependence, is presented in Figure 6 and Figure 7.

It should be noted that when two different sources and technologies provide similar data, some mismatch is expected. However, any mismatches involved in the fusion of the datasets are approximated. In this case, the two datasets have an important correlation of 71%. Nevertheless, due to the wider coverage of satellite information and the lower resolution, it was applied as auxiliary information. Obviously, the fusion of the datasets in terms of STRK requires a significant correlation between the datasets.

STRK can be also applied with data in shorter time scales (e.g., daily and monthly), which can be more useful for agricultural and hydrological purposes. This work validates the applicability of STRK while exploiting the correlated satellite and geographical auxiliary information so it can be extended in the future in different time scales and applications. However, the processing of large-size datasets using STRK may be time-consuming during the data interdependence calculations and in the estimation procedure.

The generated precipitation map of the island of Crete shows a spatial distribution that agrees with previous findings of other works in the area, e.g., the precipitation gradient from east to west and the low precipitation rates at the south parts of the island. In comparison to other important similar works in the area of Crete [23,40,53,54,55] using similar dataset distribution, the spatiotemporal approach provides significantly improved results and a significantly reduced uncertainty of estimations. Specifically, the proposed model improves the estimation error on average 40% and reduces the estimation uncertainty by 25%. In addition, the results of the best-performing method competes with those of recent similar applications that apply STRK [13,28,31,33]. All of these works provide similar estimation errors of around 10–15% in terms of the mean absolute relative error metric. The developed approach compared to similar methodological applications successfully employs satellite precipitation data as a covariate which has not been widely applied in the spatiotemporal framework, but mainly in a solely spatial or temporal context. In addition, the employment of a geographical parameter (e.g., easting direction) as a covariate that explains the spatial distribution of precipitation in terms of a specific axis is applied for the first time. Overall, the combination of this information with a trend model represents a novel approach in STRK method application that improves spatial resolution and estimation accuracy. Furthermore, this work validates the applicability of the sum-metric space–time variogram in precipitation data analysis using space–time geostatistics.

## 4. Conclusions

The aim of this research was to introduce a space–time approach for predicting precipitation on the island of Crete based on STRK and auxiliary information from existing satellite data obtained with machine learning processing and geographical features. This study of blending satellite and ground precipitation observations using geostatistics and machine learning output shows the advantages of this approach compared to previous applications in the area. The major outputs of this work are: (a) the successful involvement of a trend model that comprises satellite precipitation data and the spatial distribution of observed precipitation in terms of a specific axis—in this case the easting coordinate direction—in space–time geostatistical analysis of precipitation variations using STRK; (b) the successful employment of the joint space–time data interdependence in terms of the sum-metric function which strengthened the efficiency of the spatiotemporal process; and (c) the improved results compared to previous works in the study area and to similar space–time approaches.

The proposed geostatistical model provides accurate and reliable results and can act as a good, useful and reliable tool for the spatiotemporal analysis of hydrometeorological variables in other study areas. It could be applied effectively in areas with similar properties and data availability, exploiting different geographical or geomorphological properties depending on the case study, such as elevation, distance from the sea, topographical index etc., and it will be especially valuable in semi-arid areas prone to climate change to assess effects on hydrometeorological variables variations.

Future research plans consider the involvement of more auxiliary satellite variables to the space–time model such as temperature, Normalized Difference Water Index (NDWI) or Normalized Difference Vegetation Index (NDVI) to study different hydrological processes in the island of Crete. Further validation of our approach by comparing it with other interpolation techniques with a similar concept will be carried out. Finally, the exploration of non-stationary modeling tools in order to cope with varying complexity of the terrain and its influence on precipitations is a promising avenue for research.

## Figures and Tables

**Table 1:** Estimation error of the different methods applied with the available dataset to estimate the annual precipitation values for the hydrological year 2016/17. MAE stands for mean absolute error, MARE for mean absolute relative error, and bias is the difference between observations and estimations.

Method	MAE (mm)	MARE %	Bias (mm)
Space–time residual kriging/sum-metric variogram	54.30	0.11	−22.13
Space–time residual kriging/product-sum variogram	59.65	0.12	−25.14

**Table 2:** Sum-metric variogram models and paraments.

Model	Sill	Range (Correlation Length)	Nugget	*α*
Spatial Gaussian	36.31 mm^2^	0.25 or 70 km	5.25 mm^2^	
Temporal Exponential	38.16 mm^2^	4 y	0	
Joint Exponential	37.24 mm^2^	1.14	0	0.95

## Data Availability

The source meteorological stations data in the island of Crete can be downloaded from https://www.meteo.gr/crete/ (accessed on 30 April 2021). The processed satellite precipitation data from the PERSIANN-CCS database for the island of Crete are available from the authors’ upon request.
